# Could Microparticles Be the Universal Quality Indicator for Platelet Viability and Function?

**DOI:** 10.1155/2016/6140239

**Published:** 2016-12-08

**Authors:** Elisabeth Maurer-Spurej, Kate Chipperfield

**Affiliations:** ^1^LightIntegra Technology Inc., 650-999 West Broadway, Vancouver, BC, Canada V5Z 1K5; ^2^Canadian Blood Services, Centre for Blood Research, 2350 Health Sciences Mall, Vancouver, BC, Canada; ^3^Department of Pathology and Laboratory Medicine, University of British Columbia, Vancouver, BC, Canada; ^4^Hematopathology, Department of Pathology and Laboratory Medicine, British Columbia Children's Hospital, 4480 Oak Street, Room 2K49 Vancouver, BC, Canada V6H 3V4

## Abstract

High quality means good fitness for the intended use. Research activity regarding quality measures for platelet transfusions has focused on platelet storage and platelet storage lesion. Thus, platelet quality is judged from the manufacturer's point of view and regulated to ensure consistency and stability of the manufacturing process. Assuming that fresh product is always superior to aged product, maintaining in vitro characteristics should preserve high quality. However, despite the highest in vitro quality standards, platelets often fail in vivo. This suggests we may need different quality measures to predict platelet performance after transfusion. Adding to this complexity, platelets are used clinically for very different purposes: platelets need to circulate when given as prophylaxis to cancer patients and to stop bleeding when given to surgery or trauma patients. In addition, the emerging application of platelet-rich plasma injections exploits the immunological functions of platelets. Requirements for quality of platelets intended to prevent bleeding, stop bleeding, or promote wound healing are potentially very different. Can a single measurable characteristic describe platelet quality for all uses? Here we present microparticle measurement in platelet samples, and its potential to become the universal quality characteristic for platelet production, storage, viability, function, and compatibility.

## 1. Introduction

High quality performance is achieved when the best tool or process is employed for the intended use. Currently, with the end-goal of high quality platelet products for transfusion, platelet concentrate production, manipulation, and storage are tailored to maintaining platelet viability. Anticoagulation, consistency, and stability of the manufacturing process, limited exposure to stress, and optimal storage conditions are tightly controlled parameters to preserve platelet viability in vitro and prevent degradation, also known as platelet storage lesion. It is assumed that most donors donate viable platelets and that viability is lost due to the storage lesion; donor variability is not considered a major contributing factor [1]. It then follows that patients needing viable platelets that remain in circulation for some time would benefit most from the freshest product. Traditionally, in vitro platelet quality measures have been based on these assumptions [2]. Parameters like CD62 expression, response to ADP, or hypotonic shock are measured because there is a physiological rationale behind changes in these measures that occur with both activation and aging of platelets. Platelet release of microparticles has also been shown to follow platelet activation and increase with aging of platelet products [3]. Thus, platelet quality is assessed from the manufacturer's point of view and regulated to ensure consistency and stability of the manufacturing process [4].

Because the emphasis is to detect degradation from the beginning to the end of the current 5-day shelf life [5, 6], the resolution of current quality measures has been tuned to be high for small changes on the “resting/viable” end of the quality spectrum but becomes low on the “activated/functional” end of the quality spectrum. Additional changes are seen upon extended shelf life which become more important as bacterially tested or pathogen inactivated platelets may be stored for 7 days. Some sensitive markers start to fail if the storage lesion effects are too big. However, correlation between different in vitro tests improves with the inclusion of data outside the highly variable normal range [7]. It is therefore not surprising that these measures are often poor predictors of clinical outcome [8] and that studies do not consistently find that in vitro parameters correlate well with in vivo outcome [8–11]. Irrespective of product age, we observed that 17% (1 in 6) of platelet transfusions did not produce the expected posttransfusion platelet count increments in hematology-oncology patients [12]. One possible cause of this unexpectedly high rate of poor clinical outcome is that platelet viability is highly influenced by donor characteristics. Indeed, approximately 33% of normal donors donate preactivated platelets as indicated by high microparticle levels [13]. Cancer patients typically need platelets not because they are actively bleeding, but because they are at risk of bleeding due to low platelet counts secondary to disease and/or therapy. In these patients, transfused platelets are required to stay in circulation, ready to respond to nontraumatic microvascular bleeding. Due to their reduced viability, preactivated platelets are not recommended for storage or for prophylaxis in cancer patients [14]. In contrast, preactivated platelets are thought to be beneficial when immediate haemostatic function is required to stop acute bleeding, particularly important for surgical or trauma patients [15].

Platelets are used clinically for very different purposes and platelet quality must be compatible with the respective purpose of transfusion. Consequently, the ideal quality parameter to predict platelet performance after transfusion must be able to differentiate between resting/viable and preactivated/highly functional platelets with similar resolution across the entire viability-functionality spectrum.

Activated platelets shed microparticles [16], and platelets are the major source of circulating microparticles [17, 18]. In this review, we explore the possibility that microparticle content as a measure of platelet fragmentation and heterogeneity may fulfill the requirement for a universal quality indicator for platelet production, storage, viability, function, and compatibility [19–21]. We assess the heterogeneity of platelet concentrates from the three different perspectives of production, prophylactic transfusion, and therapeutic transfusion.

## 2. Platelet Quality from the Perspective of Production

### 2.1. Platelet Quality Measures

In vitro measures have been based on the assumption that fresh platelets are better than old platelets. Thus, discoid platelets with low expression of CD62 [22] and other activation markers and low release of intracellular or metabolic substances are deemed high quality, whereas the opposite is deemed poor quality [23]. The panel of in vitro tests also includes functional measures such as the response of platelets to ADP or hypotonic shock [24, 25].

### 2.2. Homogeneous Compared to Heterogeneous Platelets

**Figure 1:**
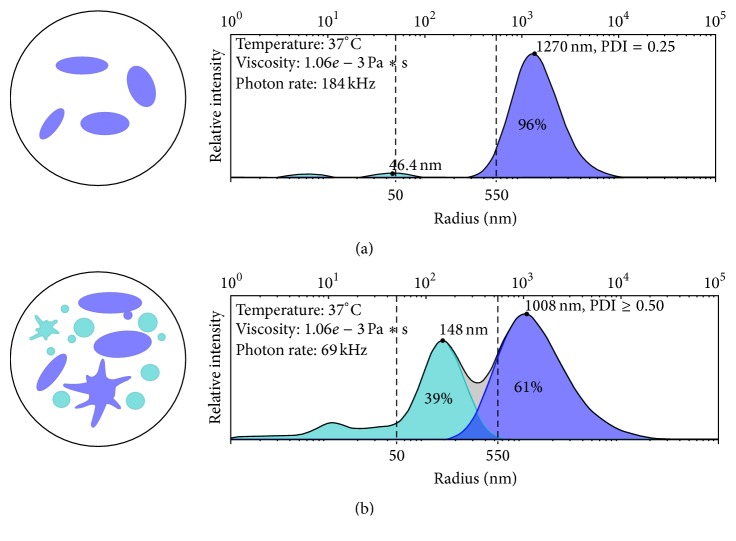
Example of dynamic light scattering test results showing the contribution of exosome-sized particles (radii below 50 nm), microparticles (radii 50–550 nm), platelets, and microaggregates (radii above 550 nm). (a) Homogeneous platelets (few or no microparticles, platelets with predominantly discoid shape with low polydispersity, and narrow blue peak). (b) Heterogeneous platelets (many microparticles, platelets with high polydispersity, and broad blue peak).

If platelets are more viable—that is, fit to survive storage, transportation, gamma irradiation, and other processes that might happen prior to transfusion—then they are by definition less functional. This has been known since the 1970s when investigators sought to define the right storage temperature for platelet concentrates. Several research teams reported that preservation of platelets for storage and optimal radiolabel recovery and survival caused a reversible dysfunction of platelets' hemostatic capability. In short, room temperature stored platelets were more viable but refrigerated platelets showed better function assessed by increased haemostasis in aspirin-treated volunteers or thrombocytopenic patients [26, 27]. Keeping platelets viable is an important role of anticoagulants. Concentrates rich in viable platelets are homogeneous in their composition, containing primarily discoid platelets and few or no microparticles or microaggregates (Figure 1(a)) [13].

In contrast, aged, chilled, or otherwise activated platelets are expected to be heterogeneous, containing platelets with high polydispersity due to a large spread of different morphologies, high surface expression of activation markers, many microparticles, and the presence of microaggregates [21, 28, 29] (Figure 1(b)). Heterogeneous platelets are known to be more functional but less viable. This is shown in animal experiments where heterogeneous platelets do not survive long in circulation, particularly in animals with inflammatory conditions [30]. Considering that aged platelets are often heterogeneous, these platelets are likely primed for cell death [31].

### 2.3. Microparticles as Quality Indicators

The heterogeneity of platelet concentrates increases with storage [10, 13, 32, 33] and with pathogen-reduction processing [5, 13] and varies greatly between normal donors [13, 34–36]. The largest contributor to platelet heterogeneity is the microparticle content (Figure 1(b)). Microparticles, also known as extracellular vesicles, are abundant in certain platelet concentrates where microparticles contain extracellular mitochondria [37, 38]. Microparticles are implicated as a transport and delivery system of mediators participating in hemostasis, thrombosis, vascular repair, and inflammation, acting both locally and systemically under physiologic as well as pathophysiologic conditions [17, 39–41]. Microparticles express membrane-associated proteins and are able to transfer receptors, growth factors, and microRNA between cells [32, 39, 40, 42]. If microparticles contain mitochondria they might be associated with adverse inflammatory reactions in recipients [38]. Many of the details of the origin and composition of microparticles are under investigation and, due to the limitations of some testing systems, results may be controversial [43].

Two important questions have long been proposed for investigation: first, whether microparticles in blood products have a potentially pathogenic effect, and second, how blood product processing and storage affect microparticle release [32]. More recently, regulatory agencies are recognizing the importance of microparticles as quality indicators due to their potential physiological and pathophysiologic roles. The US Food and Drug Administration acknowledged the importance of microparticles in transfusion medicine because microparticles are present in both plasma and cellular blood products [32]. Finally, Paul-Ehrlich Institute in Germany licensed ThromboLUX (Table 1) microparticle testing for use in transport validation.

### 2.4. Assessment of Microparticles in Blood

Several well-established research technologies have been used and described in the literature for the measurement of microparticles in blood and other body fluids (Table 1), including dynamic light scattering (DLS) as used in ThromboLUX (LightIntegra Technology Inc.) [13, 44], flow cytometry (FC) [37], and ELISA [45, 46]. The optical system, small sample volume, and specific software used in ThromboLUX address the challenges other DLS instruments face with testing platelet-rich plasma or platelet concentrates [20, 43]. The qNano Gold (Izon Science) uses size exclusion chromatography and resistive pulse sensing [47], and a combination of dynamic light scattering and particle tracking is used by NanoSight (Malvern) [48]. Excellent recent reviews describe all but the latest dynamic light scattering testing methods and how they can be used in various biological media including blood [49, 50].

### 2.5. Limitations of Microparticle Tests

Here we review studies on the limitations of currently available microparticle tests (Table 2). It is generally recognized that accurate determination of microparticle concentration with flow cytometric methods is problematic [51–53]. Flow cytometric methods have limitations in cases where microvesicles form aggregates or complexes with each other or with cells. In comparison, dynamic light scattering assays are not designed as whole blood assays and therefore require centrifugation to obtain PRP. Large aggregates present in whole blood would be removed during centrifugation but aggregates that formed in concentrates over time or with certain product manipulations would result in a broadening of the platelet population and increase of the polydispersity index for platelets. If a microparticle assay does not require ultracentrifugation, artifacts from sample preparation are unlikely, and the assay can be conducted in the presence of platelets and other particles. Microparticles and chylomicrons are differentiated neither by scattering-based flow cytometry or dynamic light scattering in platelet-rich plasma, nor by nanoparticle tracking analysis (NTA) or tunable resistive pulse sensing (TRPS) in platelet-poor plasma [54], which will affect accurate cell-derived microparticle quantification in lipid-rich samples.

Use of flow cytometry could lead to an underestimation of microparticle content if smaller microparticles are not counted [50, 55, 56]. Flow cytometry is capable of analyzing platelet microparticles <1 micron in size in plasma sources and may be more accurate than ELISA, which may fail to immobilize platelet microparticles >100 nm in diameter [55]. Smaller microparticles are not detected by standard flow cytometry because they are excluded when the operator cuts out electronic noise by setting thresholds [57], but high-sensitivity flow cytometry (hs-FCM) can now discriminate previously undetectable small microparticles in plasma samples [56]. In contrast to flow cytometry, underestimation of microparticle content is not relevant to dynamic light scattering-based assays, which provide qualitative and quantitative information for microparticles/extracellular vesicles in the radius range of 1 nm to 550 nm. However, DLS-based assays do not differentiate between cellular fragments shed from platelets, red blood cells, white blood cells, or endothelial cells. The NanoSight can differentiate the cellular origin of microparticles based on its fluorescence capability. However, the limitations of this technique as described in the operator's manual—chamber leaks, bubbles, and the risk of contamination from cleaning and reusing the chamber—suggest that this method may not be conducive to routine use.

Selecting the right probe or marker for microparticle detection is another important issue. Annexin V is widely used in flow cytometry to select entire microparticle populations based on binding to exposed phosphatidylserine. Calcium- dependent binding can result in plasma clotting, but a modification using heparin was successfully tested [58]. When targeting Annexin V for microparticle detection, loss of sensitivity occurs when phosphatidylserine levels are low. Whether all microparticles bind Annexin V and whether a high concentration of Annexin V-binding microparticles relates to poor viability (poor posttransfusion recovery) are still unanswered questions.

Standardization is an unresolved issue in microparticle detection. It is now well accepted that accurate bead standards with appropriate refractive indices to gate microparticles by flow cytometry still need to be developed [47, 59]. Currently, comparing data from different studies is difficult due to the wide variety of methods for microparticle determination used by different laboratories [60]. Dedicated instruments configured to perform microparticle screening have the advantage of reduced assay-to-assay and system-to-system variability. Method-to-method standardization is also being investigated in other systems. For example, the clot-based procoagulant phospholipid assay correlates significantly with a thrombin generation assay [61] and the study authors suggest that the thrombin generation assay may be the more sensitive measure for procoagulant activity of microparticles carrying active tissue factor.

Methods for microparticle detection show good correlations of results, although comparability of counts by flow cytometry and microparticle activity may be limited due to different assay principles [62]. Counts are based on detecting the intensity of scattered or fluorescent light as microparticles move through the laser beam of a flow cytometer while microparticle activity tests rely on chemical reactions of microparticle components. Relative platelet microparticle counts measured by flow cytometry were shown to strongly correlate with the microparticle content measured by one dynamic light scattering assay in both platelet-rich plasma and apheresis platelet concentrates [20, 44]. However, when the reported relative microparticle content is converted to concentrations, the numbers obtained by dynamic light scattering are 100–1000 times higher than those reported by others (Table 2). This is possibly due to the use of native or fixed samples without differential centrifugation to remove platelets prior to testing. The much lower microparticle concentrations detected by flow cytometry could be related to (1) beads being inadequate as size standards [47], (2) loss of microparticles below the electronic threshold [44], and (3) limitations such as swarm detection [57, 59].

Currently there is no consensus on the best measures for accuracy of microparticle concentration values, size detection, probe selection, standardization, or appropriateness for testing of specific samples, and research is ongoing. Methods such as ThromboLUX cannot be used to characterize various microparticle subpopulations; however, they do allow routine microparticle screening of platelet concentrates at various points of the product life cycle [12, 20, 44].

## 3. Platelet Quality Measures for Prophylactic Transfusion

### 3.1. Platelet Viability

We observed that ThromboLUX-measured microparticle content in fresh, normal-donor platelet-rich plasma was inversely associated with radiolabeled platelet recovery in autologous transfusions (unpublished results). The mechanism of how microparticles could reduce platelet recovery after reinfusion is not known. Three scenarios have been suggested: (1) microparticles might have a direct effect on the recipient's immune system, (2) the factors that generate microparticles also mark the platelets for removal from circulation, and/or (3) microparticle generation indicates platelet activation and preactivated platelets are consumed by daily vascular maintenance. In a recent publication our collaborators on this unpublished work found lipid oxidation products–which are linked to platelet activation and heterogeneity–to be associated with poor posttransfusion performance [63]. If homogeneous autologous platelet transfusions give better recovery it might be expected that patients receiving allogeneic transfusions for prophylaxis would also benefit when platelets are homogeneous. Here it is suggested that homogeneous, viable platelets give better recovery measured as count increments.

### 3.2. Platelet Refractoriness and Platelet Compatibility

Platelet refractoriness, a situation in which the patient does not show the expected response to the platelet transfusion [64], is a complication seen in up to 27% of platelet recipients [65]. Platelet refractoriness is defined as two consecutive platelet transfusions resulting in insufficient corrected (platelet) count increments (CCI). The threshold below which a CCI is deemed insufficient depends on the time point of measurement: a CCI less than 5,000–7,500 platelets/*μ*L measured in the recipient's blood sample drawn 1 hour after transfusion characterizes poor recovery; a CCI less than 5,000 platelets/*μ*L in a sample drawn 24 hours after transfusion characterizes poor survival. Patients with immune refractoriness show low posttransfusion CCI at both 1 hour and 24 hours after transfusion which may or may not be addressed with HLA/HPA matched platelet concentrates [65, 66]. However, often, even when there is no documented alloimmunisation, the 1-hour platelet increment is satisfactory followed by a significant decrease in platelet count at 24-hour posttransfusion. Poor platelet quality was suggested as one reason why transfused cancer patients may show especially poor platelet survival at 24 hours [12]. In addition to the impact on patient care, inpatient hospital costs for a platelet-refractory patient (approximately US$ 104,000) are more than double compared to nonrefractory patients, with hospital stays 21 days longer [67].

Microparticles are prothrombotic inflammatory markers. Patients who become refractory to platelet transfusion often have concurrent fever or systemic inflammation that might be detectable as elevated microparticles [68, 69]. Homogeneous platelets may therefore be the best choice for cancer patients at risk for platelet refractoriness while heterogeneous platelets may be incompatible with patients challenged by preexisting inflammation. It follows that platelet transfusions from donors with high microparticle content may only be compatible with patients without preexisting inflammation. It is conceivable that transfusing heterogeneous platelets to patients whose immune systems are more activated can push them to the tipping point to become platelet-refractory. Avoiding transfusion of heterogeneous platelets for prophylactic use might prevent refractoriness. Interestingly, a very similar two-hit concept has been suggested for red blood cell transfusions based on dog studies: dogs with bacterial infection (first hit) receiving older red blood cells containing higher concentrations of microparticles (second hit) had a much higher risk of mortality [70, 71].

## 4. Platelet Quality for Therapeutic Use

### 4.1. Platelet Hemostatic Function

Heterogeneous platelet concentrates contain preactivated platelets, which are fit to react quickly once they enter circulation [15, 21, 28]. Thus, heterogeneous platelets are highly functional and have been shown to stop bleeding faster than homogeneous, viable platelets [28].

## 5. Platelet Quality for Platelet-Rich Plasma Injections

Platelet-rich plasma (PRP) injections are currently not managed by blood operators because they are autologous products: patients are phlebotomized of a small volume of whole blood which is then processed and reinjected to treat a variety of conditions including chronic tendon injuries, osteoarthritis, and bone regeneration. There are no clear quality standards for PRP injections and the current existing variability has been described previously [72]. The mechanism by which PRP injections exert their healing properties is still not known but the abundance of growth factors present in platelets [73] and the bactericidal and other immune functions platelets possess [74] are thought to play a major role. In the context of this review it is conceivable that microparticles also play a role in these autologous treatments. Samples from patients who already suffer from systemic inflammation might show microparticles as indicators of systemic inflammation and thus be predictors of a reduced likelihood of treatment success. On the other hand, it has been suggested that microparticles are carriers of growth factors and might significantly contribute to the healing properties of PRP [75]. This is an area that requires further study.

## 6. Future Direction

Implementation of microparticle measurements for quality control of platelet concentrates could address the impact of pathogen inactivation, platelet additive solutions, and 7-day storage. Inventory management based on this measure could lead to optimization of patient care and reduce cost at the same time.

Platelet viability would best be described in terms of posttransfusion platelet recovery at 24 hours, which is inversely associated with microparticle content. Clinical studies are needed to confirm our currently unpublished pilot data as well as the animal experiments that seem to support the hypothesis that patients with existing inflammation (first hit) receiving a transfusion of heterogeneous platelets (second hit) have a high risk of becoming refractory.

## 7. Conclusion

The technology for testing of microparticle content as a marker of the heterogeneity of platelet concentrates is a developing field. The selection of the most appropriate method of measurement for each situation remains to be determined. However, the compatibility of homogeneous versus heterogeneous platelet concentrates for clinical use is becoming clear. For prophylaxis, giving homogeneous, viable platelets to cancer patients should be advantageous because these are expected to circulate and not be immediately removed from circulation. For use as a therapeutic agent to stop bleeding, concentrates rich in heterogeneous platelets might react better, as was shown with chilled and preactivated platelets. Thus, by measuring the composition of a platelet concentrate, the performance of the concentrate during storage and its resilience to additional stress could be determined and inform its optimal use.

Implementation of routine screening of platelet concentrates requires a quick and easy, noninvasive test that measures platelet characteristics meaningful to all aspects of platelet quality. We have proposed microparticle content to be that characteristic parameter. The FDA recommends determination of platelet microparticle content but until recently there was no quick and easy test method to achieve this. Some technological challenges of dynamic light scattering have been resolved with ThromboLUX, which does not characterize microparticle subpopulations and as such is not an in-depth research tool but allows routine microparticle screening of platelet-rich plasma or platelet concentrates.

Potential new research could address the reason for the heterogeneity of platelet donors, ways to influence the subsequent heterogeneity of the donated product, which could either decrease the heterogeneity for prophylactic use, for example, by nanofiltration [76], or increase the heterogeneity for therapeutic use, for example, by chilling. Finally, the clinical and health economic impact of platelet quality determination and subsequent inventory management warrants investigation.

## Figures and Tables

**Table 1:** Comparison of microparticle testing technologies.

Technology	Principle	Manufacturer	Invasive^1^	Standards, dilutions required	Trained specialist required	MP separation required	Prep. time [min]	Time/test [min]	Daily maintenance
ThromboLUX	DLS	LightIntegra	No	No	No	No	5	8	No

Flow cytometer	Static light scattering, fluorescence	Abbott, Becton Dickinson amongst others	No	Yes	Yes	Yes	30+	5–10	Yes

Micro flow cytometer	Static light scattering, fluorescence	Apogee flow systems	No	Yes	Yes	Yes	30+	5–10^*∗*^	Yes

qNano Gold	Size exclusion chromatography	Izon Science	Yes	Yes	Yes	Yes	20^†^	10–15	No

NanoSight	DLS particle tracking	Malvern	Yes	Yes	Yes	Yes	15	6–75	No

ELISA	Double antibody sandwich technique	JIMRO Co. Ltd., Diagnostica Stago	No	Yes	Yes	Yes	30+	5–10	No

^1^The test is considered invasive if the required sample volume is larger than what can be aseptically obtained from a tubing segment.

^*∗*^Consumables only usable for 6–8 hours. ^†^Isolation of microparticles by differential centrifugation or size exclusion chromatography required, dynamic light scattering (DLS), microparticles (MP), and enzyme-linked immunosorbent assay (ELISA).

**Table 2:** Overview of published clinical microparticle studies.

Performance topic	Reference	Type of microparticle assay	Total number of subjects in study	Concentration [MP/L]	Summary statement
Accurate enumeration of microparticles (especially in the presence of platelets or other particles)	Balvers et al. 2015 [51]	FC	20 (10 trauma patients; 10 healthy)	7.5 × 10^3^	Flow cytometry does not count microparticles if bound in complexes; reported concentration is about 10^6^ lower than reported elsewhere; sample was prepared at low temperature
	Jayachandran et al. 2011 [52]	FC	118 (58 assayed for plasma microparticles)	N/A	Flow cytometry does not detect aggregates
	van Ierssel et al. 2012 [53]	FC	13 in vitro lipid (5 coronary heart disease; 8 healthy); 5 in vivo lipid, healthy	2.5 × 10^8^ (EMP only)	Flow cytometry data are affected by high circulating levels of lipids

Size of microparticles (below the detection limit of many technologies)	Leong et al. 2011 [55]	FC	6 (acute myocardial infarction; healthy)	3 × 10^9^	Platelet microparticle size is below stated detection limits of most flow cytometers. However, study confirmed that flow cytometry is capable of analyzing microparticles from plasma; approximately 2-fold for acute myocardial infarction (AMI) patient
	Robert et al. 2012 [56]	FC	40 (30 coronary disease; 10 healthy)	2.0 × 10^9^ (1.1 × 10^10^ with high sensitivity FCM)	Standard flow cytometry does not detect small microparticles. High-sensitivity flow cytometry allows measurement of previously undetectable microparticles; approximately 10-fold for coronary patients

Probe/marker selection	Hou et al. 2011 [77]	FC	20 healthy donors	1 × 10^9^ (fresh) 1.5 × 10^10^ (day 9)	Annexin V does not bind to membranes at low phosphatidyl-serine levels and is Ca^2+^ dependent; lactadherin is proposed as an alternative
	Iversen et al. 2013 [58]	FC	49 (20 healthy; 29 systemic lupus erythematosus)	9 × 10^9^	Annexin V binding is Ca^2+^ dependent, resulting in potential clotting of plasma; approximately 2-fold for patients with systemic lupus erythematosus (SLE)
	Lanuti et al. 2012 [78]	FC	34 (20 diabetes; 14 healthy)	1.1 × 10^8^ (EMP only)	Endothelial microparticles and circulating endothelial cells share markers such as CD144 and CD146 leading to overestimation; approximately 2-fold for patients with type 2 diabetes (Iversen et al. published endothelial microparticle concentration to be a factor 10 lower than platelet microparticles)
	Bohling et al. 2012 [45]	ELISA, clot-based and chromogenic and flow cytometry	75 (24 healthy, 28 trauma, 23 nontrauma (patients taking warfarin, heparin, or lupus anticoagulants))	4 × 10^10^	The performance characteristics of a clot-based versus chromogenic procoagulant phospholipid assay were compared and low correlation found; neither assay was considered optimal

Standardization of methods	Marchetti et al. 2014 [61]	ELISA, clot-based and thrombin generation	145 (72 control, 73 essential thrombocythemia)		The performance characteristics of clot-based procoagulant phospholipid assay and thrombin generation assay were compared

Method selection	Strasser et al. 2013 [62]	FC, prothrombinase ELISA, clot-based ELISA	31 healthy donors	1.2 × 10^9^	The performance characteristics of a clot-based procoagulant phospholipid assay, prothrombinase assay, and flow cytometry were compared
	Labrie et al. 2013 [20]	DLS	24 apheresis platelet concentrates from normal volunteers	1.5 × 10^12^	ThromboLUX microparticle assay was compared to flow cytometry and correlated highly
	Xu et al. 2011 [44]	DLS	160 (81 platelet-rich plasma, 79 apheresis platelet concentrates)	2 × 10^11^	ThromboLUX microparticle assay was compared to flow cytometry [51]; values were calculated from reported relative content but concentrations are not published

Flow cytometry (FC) and dynamic light scattering (DLS).

## References

[1] Mittal K., Kaur R. (2015). Platelet storage lesion: an update. *Asian Journal of Transfusion Science*.

[2] Holme S. (1998). Storage and quality assessment of platelets. *Vox Sanguinis*.

[3] Pienimaeki-Roemer A., Kuhlmann K., Böttcher A. (2015). Lipidomic and proteomic characterization of platelet extracellular vesicle subfractions from senescent platelets. *Transfusion*.

[4] Farrugia A., Vamvakas E. (2014). Toward a patient-based paradigm for blood transfusion. *Journal of Blood Medicine*.

[5] Johnson L., Schubert P., Tan S., Devine D. V., Marks D. C. (2016). Extended storage and glucose exhaustion are associated with apoptotic changes in platelets stored in additive solution. *Transfusion*.

[6] Johnson L., Hyland R., Tan S. (2016). In vitro quality of platelets with low plasma carryover treated with ultraviolet C light for pathogen inactivation. *Transfusion Medicine and Hemotherapy*.

[7] Heaton W. A., Sweeny J. D., Lazano M. (2013). In-vivo and in-vitro evaluation of stored platelet products. *Platelet Transfusion Therapy*.

[8] Murphy S. (2004). Utility of in vitro tests in predicting the in vivo viability of stored PLTs. *Transfusion*.

[9] Goodrich R. P., Li J. Z., Pieters H., Crookes R., Roodt J., Heyns A. D. P. (2006). Correlation of in vitro platelet quality measurements with in vivo platelet viability in human subjects. *Vox Sanguinis*.

[10] Apelseth T. O., Hervig T. (2007). In vitro evaluation of platelet concentrates during storage: platelet counts and markers of platelet destruction. *Transfusion and Apheresis Science*.

[11] Apelseth T. O., Bruserud Ø., Wentzel-Larsen T., Hervig T. (2010). Therapeutic efficacy of platelet transfusion in patients with acute leukemia: an evaluation of methods. *Transfusion*.

[12] Maurer-Spurej E., Labrie A., Pittendreigh C. (2009). Platelet quality measured with dynamic light scattering correlates with transfusion outcome in hematologic malignancies. *Transfusion*.

[13] Maurer-Spurej E., Larsen R., Labrie A., Heaton A., Chipperfield K. (2016). Microparticle content of platelet concentrates is predicted by donor microparticles and is altered by production methods and stress. *Transfusion and Apheresis Science*.

[14] Schiffer C. A., Anderson K. C., Bennett C. L. (2001). Platelet transfusion for patients with cancer: clinical practice guidelines of the American Society of Clinical Oncology. *Journal of Clinical Oncology*.

[15] Getz T. M., Montgomery R. K., Bynum J. A., Aden J. K., Pidcoke H. F., Cap A. P. (2016). Storage of platelets at 4°C in platelet additive solutions prevents aggregate formation and preserves platelet functional responses. *Transfusion*.

[16] Ripoche J. (2011). Blood platelets and inflammation: their relationship with liver and digestive diseases. *Clinics and Research in Hepatology and Gastroenterology*.

[17] Flaumenhaft R. (2006). Formation and fate of platelet microparticles. *Blood Cells, Molecules, and Diseases*.

[18] Keuren J. F. W., Magdeleyns E. J. P., Govers-Riemslag J. W. P., Lindhout T., Curvers J. (2006). Effects of storage-induced platelet microparticles on the initiation and propagation phase of blood coagulation. *British Journal of Haematology*.

[19] Johnson L., Reade M. C., Hyland R. A., Tan S., Marks D. C. (2015). In vitro comparison of cryopreserved and liquid platelets: potential clinical implications. *Transfusion*.

[20] Labrie A., Marshall A., Bedi H., Maurer-Spurej E. (2013). Characterization of platelet concentrates using dynamic light scattering. *Transfusion Medicine and Hemotherapy*.

[21] Johnson L., Tan S., Wood B., Davis A., Marks D. C. (2016). Refrigeration and cryopreservation of platelets differentially affect platelet metabolism and function: a comparison with conventional platelet storage conditions. *Transfusion*.

[22] Levin E., Serrano K., Devine D. V. (2013). Standardization of CD62P measurement: results of an international comparative study. *Vox Sanguinis*.

[23] Maurer-Spurej E., Chipperfield K. (2007). Past and future approaches to assess the quality of platelets for transfusion. *Transfusion Medicine Reviews*.

[24] Holme S. (2008). In vitro assays used in the evaluation of the quality of stored platelets: correlation with in vivo assays. *Transfusion and Apheresis Science*.

[25] Akay O. M., Gündüz E., Başyiğit H., Gulbas Z. (2007). Platelet function testing during 5-day storage of single and random donor plateletpheresis. *Transfusion and Apheresis Science*.

[26] Valeri C. R. (1976). Circulation and hemostatic effectiveness of platelets stored at 4 C or 22 C: studies in aspirin-treated normal Volunteers. *Transfusion*.

[27] Becker G. A., Tuccelli M., Kunicki T., Chalos M. K., Aster R. H. (1973). Studies of platelet concentrates stored at 22 C and 4 C. *Transfusion*.

[28] Reddoch K. M., Pidcoke H. F., Montgomery R. K. (2014). Hemostatic function of apheresis platelets stored at 4°C and 22°C. *Shock*.

[29] Neumüller J., Meisslitzer-Ruppitsch C., Ellinger A. (2013). Monitoring of platelet activation in platelet concentrates using transmission electron microscopy. *Transfusion Medicine and Hemotherapy*.

[30] Lu Y., Li Q., Liu Y.-Y. (2015). Inhibitory effect of caffeic acid on ADP-induced thrombus formation and platelet activation involves mitogen-activated protein kinases. *Scientific Reports*.

[31] Mason K. D., Carpinelli M. R., Fletcher J. I. (2007). Programmed anuclear cell death delimits platelet life span. *Cell*.

[32] Simak J., Gelderman M. P. (2006). Cell membrane microparticles in blood and blood products: potentially pathogenic agents and diagnostic markers. *Transfusion Medicine Reviews*.

[33] Maurer-Spurej E., Pfeiler G., Maurer N., Lindner H., Glatter O., Devine D. V. (2001). Room temperature activates human blood platelets. *Laboratory Investigation*.

[34] Phang M., Lincz L., Seldon M., Garg M. L. (2012). Acute supplementation with eicosapentaenoic acid reduces platelet microparticle activity in healthy subjects. *Journal of Nutritional Biochemistry*.

[35] Wu S.-Y., Mayneris-Perxachs J., Lovegrove J. A., Todd S., Yaqoob P. (2014). Fish-oil supplementation alters numbers of circulating endothelial progenitor cells and microparticles independently of eNOS genotype. *American Journal of Clinical Nutrition*.

[36] Sossdorf M., Otto G. P., Claus R. A., Gabriel H. H. W., Lösche W. (2011). Cell-derived microparticles promote coagulation after moderate exercise. *Medicine & Science in Sports & Exercise*.

[37] Boilard E., Duchez A.-C., Brisson A. (2015). The diversity of platelet microparticles. *Current Opinion in Hematology*.

[38] (2015). Boudreau LH, Duchez A-C, Cloutier N, et al. Platelets release mitochondria serving as substrate for bactericidal group IIA-secreted phospholipase A2 to promote inflammation. *Blood*.

[39] Cognasse F., Hamzeh-Cognasse H., Laradi S. (2016). The role of microparticles in inflammation and transfusion: a concise review. *Transfusion and Apheresis Science*.

[40] Burnouf T., Chou M.-L., Goubran H., Cognasse F., Garraud O., Seghatchian J. (2015). An overview of the role of microparticles/microvesicles in blood components: are they clinically beneficial or harmful?. *Transfusion and Apheresis Science*.

[41] Laffont B., Corduan A., Rousseau M. (2016). Platelet microparticles reprogram macrophage gene expression and function. *Thrombosis and Haemostasis*.

[42] Goubran H. A., Burnouf T., Stakiw J., Seghatchian J. (2015). Platelet microparticle: a sensitive physiological “fine tuning” balancing factor in health and disease. *Transfusion and Apheresis Science*.

[43] Black A., Pienimaeki-Roemer A., Kenyon O., Orsó E., Schmitz G. (2015). Platelet-derived extracellular vesicles in plateletpheresis concentrates as a quality control approach. *Transfusion*.

[44] Xu Y., Nakane N., Maurer-Spurej E. (2011). Novel test for microparticles in platelet-rich plasma and platelet concentrates using dynamic light scattering. *Transfusion*.

[45] Bohling S. D., Pagano M. B., Stitzel M. R., Ferrell C., Yeung W., Chandler W. L. (2012). Comparison of clot-based vs chromogenic factor Xa procoagulant phospholipid activity assays. *American Journal of Clinical Pathology*.

[46] Nomura S., Shouzu A., Taomoto K. (2009). Assessment of an ELISA kit for platelet-derived microparticles by joint research at many institutes in Japan. *Journal of Atherosclerosis and Thrombosis*.

[47] van der Pol E., Coumans F., Varga Z., Krumrey M., Nieuwland R. (2013). Innovation in detection of microparticles and exosomes. *Journal of Thrombosis and Haemostasis*.

[48] Gercel-Taylor C., Atay S., Tullis R. H., Kesimer M., Taylor D. D. (2012). Nanoparticle analysis of circulating cell-derived vesicles in ovarian cancer patients. *Analytical Biochemistry*.

[49] Burnouf T., Goubran H. A., Chou M.-L., Devos D., Radosevic M. (2014). Platelet microparticles: detection and assessment of their paradoxical functional roles in disease and regenerative medicine. *Blood Reviews*.

[50] Foster B. P., Balassa T., Benen T. D. (2016). Extracellular vesicles in blood, milk and body fluids of the female and male urogenital tract and with special regard to reproduction. *Critical Reviews in Clinical Laboratory Sciences*.

[51] Balvers K., Curry N., Kleinveld D. J. B. (2015). Endogenous microparticles drive the proinflammatory host immune response in severely injured trauma patients. *Shock*.

[52] Jayachandran M., Litwiller R. D., Lahr B. D. (2011). Alterations in platelet function and cell-derived microvesicles in recently menopausal women: relationship to metabolic syndrome and atherogenic risk. *Journal of Cardiovascular Translational Research*.

[53] van Ierssel S. H., Hoymans V. Y., van Craenenbroeck E. M. (2012). Endothelial microparticles (EMP) for the assessment of endothelial function: an in vitro and in vivo study on possible interference of plasma lipids. *PLoS ONE*.

[54] Mork M., Pedersen S., Botha J., Lund S. M., Kristensen S. R. (2016). Preanalytical, analytical, and biological variation of blood plasma submicron particle levels measured with nanoparticle tracking analysis and tunable resistive pulse sensing. *Scandinavian Journal of Clinical And Laboratory Investigation*.

[55] Leong H. S., Podor T. J., Manocha B., Lewis J. D. (2011). Validation of flow cytometric detection of platelet microparticles and liposomes by atomic force microscopy. *Journal of Thrombosis and Haemostasis*.

[56] Robert S., Lacroix R., Poncelet P. (2012). High-sensitivity flow cytometry provides access to standardized measurement of small-size microparticles-brief report. *Arteriosclerosis, Thrombosis, and Vascular Biology*.

[57] Van der Pol E., Van Gemert M. J. C., Sturk A., Nieuwland R., Van Leeuwen T. G. (2012). Single vs. swarm detection of microparticles and exosomes by flow cytometry. *Journal of Thrombosis and Haemostasis*.

[58] Iversen L. V., Østergaard O., Nielsen C. T., Jacobsen S., Heegaard N. H. H. (2013). A heparin-based method for flow cytometric analysis of microparticles directly from platelet-poor plasma in calcium containing buffer. *Journal of Immunological Methods*.

[59] Chandler W. L. (2016). Measurement of microvesicle levels in human blood using flow cytometry. *Cytometry Part B: Clinical Cytometry*.

[60] Alijotas-Reig J., Palacio-Garcia C., Llurba E., Vilardell-Tarres M. (2013). Cell-derived microparticles and vascular pregnancy complications: a systematic and comprehensive review. *Fertility and Sterility*.

[61] Marchetti M., Tartari C. J., Russo L. (2014). Phospholipid-dependent procoagulant activity is highly expressed by circulating microparticles in patients with essential thrombocythemia. *American Journal of Hematology*.

[62] Strasser E. F., Happ S., Weiss D. R., Pfeiffer A., Zimmermann R., Eckstein R. (2013). Microparticle detection in platelet products by three different methods. *Transfusion*.

[63] Zimring J. C., Slichter S., Odem-Davis K. (2016). Metabolites in stored platelets associated with platelet recoveries and survivals. *Transfusion*.

[64] Rebulla P. (2005). A mini-review on platelet refractoriness. *Haematologica*.

[65] Slichter S. J., Davis K., Enright H. (2005). Factors affecting posttransfusion platelet increments, platelet refractoriness, and platelet transfusion intervals in thrombocytopenic patients. *Blood*.

[66] Hess J. R., Trachtenberg F. L., Assmann S. F. (2016). Clinical and laboratory correlates of platelet alloimmunization and refractoriness in the PLADO trial. *Vox Sanguinis*.

[67] Meehan K. R., Matias C. O., Rathore S. S. (2000). Platelet transfusions: utilization and associated costs in a tertiary care hospital. *American Journal of Hematology*.

[68] Boilard E., Nigrovic P. A., Larabee K. (2010). Platelets amplify inflammation in arthritis via collagen-dependent microparticle production. *Science*.

[69] Diehl P., Nagy F., Sossong V. (2008). Increased levels of circulating microparticles in patients with severe aortic valve stenosis. *Thrombosis and Haemostasis*.

[70] Cortés-Puch I., Remy K. E., Solomon S. B. (2015). In a canine pneumonia model of exchange transfusion, altering the age but not the volume of older red blood cells markedly alters outcome. *Transfusion*.

[71] Flegel W. A., Natanson C., Klein H. G. (2014). Does prolonged storage of red blood cells cause harm?. *British Journal of Haematology*.

[72] Cohn C. S., Lockhart E., McCullough J. J. (2015). The use of autologous platelet-rich plasma in the orthopedic setting. *Transfusion*.

[73] Textor J. (2014). Platelet-Rich Plasma (PRP) as a therapeutic agent: platelet biology, growth factors and a review of the literature. *Platelet-Rich Plasma: Regenerative Medicine: Sports Medicine, Orthopedic, and Recovery of Musculoskeletal Injuries*.

[74] Yeaman M. R., Bayer A. S. (2013). Chapter 37-antimicrobial host defense. *Platelets*.

[75] Kim E.-S., Kim J.-J., Park E.-J. (2010). Angiogenic factor-enriched platelet-rich plasma enhances in vivo bone formation around alloplastic graft material. *Journal of Advanced Prosthodontics*.

[76] Chou M.-L., Lin L.-T., Devos D., Burnouf T. (2015). Nanofiltration to remove microparticles and decrease the thrombogenicity of plasma: in vitro feasibility assessment. *Transfusion*.

[77] Hou J., Fu Y., Zhou J. (2011). Lactadherin functions as a probe for phosphatidylserine exposure and as an anticoagulant in the study of stored platelets. *Vox Sanguinis*.

[78] Lanuti P., Santilli F., Marchisio M. (2012). A novel flow cytometric approach to distinguish circulating endothelial cells from endothelial microparticles: relevance for the evaluation of endothelial dysfunction. *Journal of Immunological Methods*.

